# Single-cell metabolite annotation by tandem mass spectrometry imaging and *ab initio* molecular dynamics-based fragmentation

**DOI:** 10.1039/d5ra05470b

**Published:** 2025-09-15

**Authors:** Mateo Topalović, Ivana Marković, Vlatka Periša, Maja Lukić, Ema Pavičić, Iva Lukić, Stefan Mrđenović, Igor Lukačević, Željko Debeljak

**Affiliations:** a Josip Juraj Strossmayer University of Osijek, Department of Physics Trg Ljudevita Gaja 6 31000 Osijek Croatia; b University Hospital Centre Osijek Josipa Huttlera 4 31000 Osijek Croatia zeljko.debeljak@gmail.com; c Josip Juraj Strossmayer University of Osijek, Faculty of Medicine Josipa Huttlera 4 31000 Osijek Croatia; d Josip Juraj Strossmayer University of Osijek, Faculty of Food Technology Osijek Franje Kuhača 18 Osijek 31 000 Croatia

## Abstract

Reliable single-cell mass spectrometry (MS) imaging and metabolite annotation represent a challenge due to small cellular dimensions, small amount of desorbed materials and densely populated databases of the *m*/*z* values of endogenous compounds. To resolve these issues, a highly sensitive analytical approach was devised for metabolite annotation purposes, relying on the correspondence between the *m*/*z* values from single-cell MS/MS spectra and the *m*/*z* values of the molecular fragments calculated using *ab initio* molecular dynamics (AIMD). The approach was applied to the annotation of *m*/*z* 337.11 Da, which cellular content is increased in chronic lymphocytic leukemia (CLL). To evaluate the approach, five candidate compounds were selected by a metabolite database search using the given *m*/*z*. Matrix-assisted laser desorption/ionization ion trap-time-of-flight (MALDI IT-TOF) MS/MS spot analysis of S-nitrosoglutathione (GSNO), one of the candidate compounds, preceded single-cell MS/MS imaging: five fragments were present in the empirical and *in silico* spectra of the GSNO solution. The sensitivity of single-cell MS imaging was optimized, and MS/MS spectra were recorded for different lymphocytes containing 0–3 fragments that were present in the *in silico* spectra of three glutathione-related compounds; however, there was no match between the empirical and *in silico* spectra of the other candidate compounds. The lateral distribution of the selected fragment showed ∼3 μm shift with respect to the optical image of the lymphocyte. The novel concept developed for single-cell MS imaging enabled metabolite annotation in malignant lymphocytic clones and showed potential for metabolite annotation in other cell suspensions.

## Abstract 2

Interpretation of empirical single-cell MS/MS imaging spectra using *ab initio* molecular dynamics-based fragmentation.

## Graphical abstract

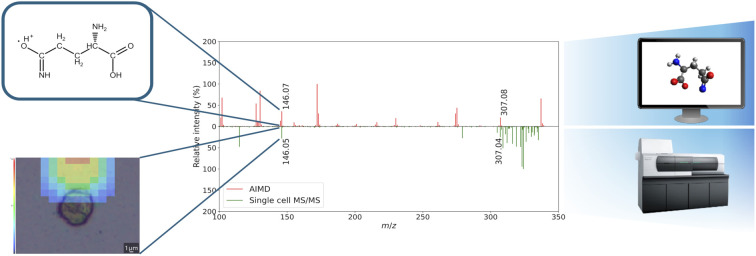

## 1 Introduction

Cellular metabolism is determined by cell differentiation, contact with the surrounding cells and tissues, blood supply and underlying health conditions. Such circumstances may lead to metabolic differences even between the neighbouring cells. Thus, single-cell metabolome heterogeneity analysis introduces additional analytical demands. Given its high degree of maturity, in most instances, LC-MS/MS technology is the first choice in metabolomics research.^1–3^ However, in the case of single-cell analysis, several obstacles hinder the application of LC-MS/MS approaches. The simplest LC-MS/MS approach involves the analysis of cell or tissue homogenates in which the information on cell-specific metabolism is lost and the sample preparation-related artefacts may be introduced. More sophisticated approaches involve either microdissection or, in the case of cell suspensions, cell sorting followed by the LC-MS/MS analysis of isolated cells.^3,4^ Unfortunately, these approaches may also introduce sample preparation-related artefacts. Besides, they require equipment that is not widely available.

Relatively simple sample preparation makes mass spectrometry imaging (MSI) a reasonable alternative to LC-MS/MS-based approaches for single-cell metabolomics. MSI, however, also suffers from some shortcomings. First of all, the small amount of material contained in a single cell raises sensitivity requirements. Furthermore, post-analytical MSI image alignment with light microscopy images may result in misalignments.^5^ Additionally, the lack of chromatographic separation accompanied by the densely populated databases of *m*/*z* signals of endogenous compounds make *m*/*z* signals extracted from the MS^1^ spectra extremely difficult to annotate using databases.^6^ Moreover, despite being densely populated, these databases still lack entries for numerous metabolites. Thus, using only MS^1^ for the annotation of metabolites is usually insufficient: MS^1^ with MS/MS may provide a more reliable annotation.^7^

Currently, at least two MSI techniques can be used for single-cell imaging: matrix assisted laser desorption/ionization time-of-flight (MALDI-TOF) MS imaging enables the analysis of a wider range of analytes in comparison to secondary ion MS imaging. Additionally, the use of MALDI-TOF MS instruments with integrated light microscopes reduces the risk of image misalignment.^5,6,8^ Although there are some recent developments in single-cell imaging,^9,10^*m*/*z* annotation remains challenging. The development of MSI instruments equipped with ion-mobility devices helps alleviate the annotation challenges.^11,12^ Furthermore, many of the already available MALDI-TOF MSI instruments can operate in MS/MS mode, which opens the possibility of fragmentation-based metabolite annotation. It is worth mentioning that the MS/MS approach for peptide identification in MSI has been successfully used for some time.^13^ Due to the fixed and relatively small number of amino acids that are linked almost exclusively by peptide bonds, peptide MS/MS spectra are relatively easy to decipher.^14^ In comparison to the MS/MS imaging of peptides, the MS/MS spectra of metabolites are much more complex to interpret, but the high cellular content of major metabolites represents an advantage for using single-cell MS/MS imaging.^15^

The problem of complex MS/MS spectra exists in other MS applications, and *in silico* approaches have emerged as possible solutions. Owing to its strong theoretical foundations, *ab initio* molecular dynamics (AIMD)-based solutions stand out.^16,17^ The stage of development of AIMD-based fragmentation enables direct comparison of *in silico* generated MS/MS spectra with the recorded single-cell MS/MS spectra beyond the scope of available reference libraries because authentic chemical standards are not provided for most molecules. This way, more reliable *m*/*z* annotation may be achieved, at least for those metabolites tentatively annotated based on their *m*/*z* values in MS^1^ spectra and available databases.^18,19^ To provide proof of the proposed metabolite annotation concept based on single-cell MALDI ion trap (IT)-TOF MS/MS imaging and AIMD-based *in silico* fragmentation, previously developed single-cell MALDI IT-TOF MS imaging of malignant lymphocyte clones^6^ was adapted for MS/MS analysis. The MS/MS spectra were then computed using an AIMD-based fragmentation method for tentatively annotated metabolites characteristic of chronic lymphocytic leukaemia (CLL), which were then compared to the single-cell MALDI IT-TOF MS/MS spectra obtained from CLL clones. The metabolites were selected on the basis of the close proximity of their *m*/*z* values (<100 ppm), *i.e.*, their overlap in MS^1^ spectra. This kind of real-world problem represents a challenging annotation task suited for this proof-of-concept study.

## 2 Materials and methods

### 2.1 Experimental design

**Fig. 1:**
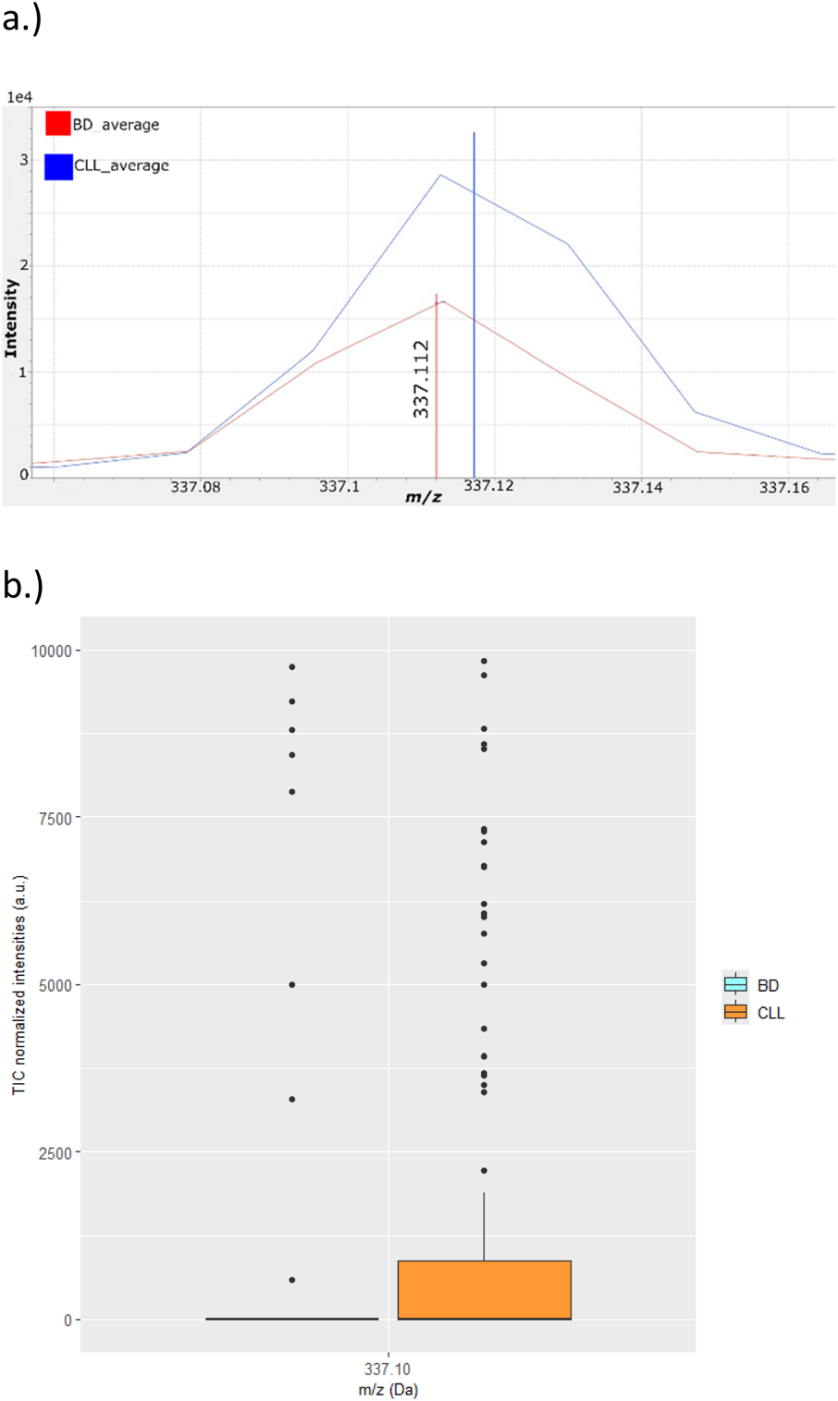
Single-cell MALDI IT-TOF MS^1^ imaging of CD19+ lymphocytes of BD and CLL participants.^6^ (a) Part of the single-cell MS^1^ spectra averaged over 2 × 200 CD19+ lymphocytes; (b) Box and Whisker plot showing the distribution of intensities. Each dot represents a single-lymphocyte intensity. The *Y*-axis has been truncated at 10 000 a.u.

To evaluate the proposed metabolite annotation concept, an *m*/*z* value of 337.11 Da, which comes from the single-cell MALDI IT-TOF MS^1^ analysis of CD19+ lymphocytes, was chosen.^6^ The increased intensity of this *m*/*z* value is characteristic of CLL (Fig. 1). The metabolite database search^18,19^ using this *m*/*z* value produced 5 possible annotations (Table 1): selection among database search results is a challenging annotation task, especially for single-cell MS imaging. It was assumed that the 337.11 Da signal comes from a compound having a single charge. However, it is worth mentioning that compounds having multiple charges may also produce this signal.

**Table 1:** Tentatively annotated metabolites^18,19^ found by the metabolome database search using the *m*/*z* 337.11 Da (±50 ppm mass tolerance)^6^

Monoisotopic *m*/*z* (Da)	Adduct type	Metabolite
337.08	M + H	GSNO
337.08	M + K	LPA8
337.10	M + Na	7,8-Dihydropteroic acid (H2Pte)
337.12	M + NH_4_-H_2_O	γ-Glutamylcysteinylserine (γ-ECS)
337.12	M + NH_4_-H_2_O	*S*-Hydroxymethylglutathione (GSHM)

Comparison between the experimental and theoretical MS/MS spectra is the basic idea behind the proposed metabolite annotation concept. Evaluation of the concept began with the AIMD-based fragmentation, which was evaluated against the empirical MS/MS spectra of the *S*-nitrosoglutathione (GSNO) solution: acceptable performance of the *in silico* approach was a prerequisite for the following single-cell MS imaging experiments. Single-cell MS imaging experiments started with the MS^1^ experiments. It was necessary to check the intensity of the single-cell signal at 337.11 Da: a strong signal at 337.11 Da originating from a single CD19+ lymphocyte was a prerequisite for the following single-cell MALDI IT-TOF MS/MS imaging. The single-cell MS/MS experiments required some adjustments of the instrumental variables which enabled sensitivity improvement and generation of distinctive product ions. Finally, the AIMD-based fragmentation spectra of 5 candidate molecules were generated and they were compared to the empirical, single-cell MS/MS spectra.

### 2.2 Sample preparation

#### 2.2.1 GSNO solution

GSNO, known for its high chemical instability and light-sensitivity, was freshly prepared prior to use. GSNO (Sigma-Aldrich, USA) was dissolved in deionized water to obtain a final concentration of 1 mM. To adjust the pH to approximately 7, a small volume of sodium hydroxide (NaOH) was added. All steps were carried out under reduced light conditions to prevent photodegradation and maintain the integrity of the nitroso compound. The prepared solution was stored in the dark at 15–25 °C, following previously reported procedures for minimizing GSNO degradation.^20^

#### 2.2.2 Isolation of CD19+ lymphocytes

CD19+ lymphocytes were isolated from the whole blood samples using an immunolabeling protocol, followed by lysis of the red blood cells, as described in a previously published study.^6^ Briefly, blood was collected from a single CLL patient: this study was conducted in accordance with the Declaration of Helsinki and approved by the Ethical Committee of the University Hospital Centre Osijek, Croatia (approval reference number: R1-9364/2022). Informed consent was obtained from the patient. One-hundred-and-fifty microliters of the whole blood was incubated with the CD19-FITC antibody (Beckton Dickinson, USA) for 20 minutes to label the B lymphocytes. Subsequently, 2 mL of the FACS lysing solution (Beckton Dickinson, USA) was added to the lysed red blood cells. The samples were then centrifuged and washed with PBS. The final content of the white blood cell suspension was adjusted according to the initial WBC count, and 100 μL was applied to an indium tin oxide (ITO)-coated glass slide using a cytocentrifuge for downstream single-cell analysis. The CD19+ lymphocytes were identified using fluorescently labeled CD19+ antibodies.

### 2.3 MALDI IT-TOF MS experiments

#### 2.3.1 MALDI IT-TOF MS/MS spot analysis of GSNO solution

The GSNO solution was applied to the ITO-coated glass slide (surface resistivity of 15–25 Ω sq^−1^; Merck, Germany) and it was left to dry completely at room temperature. The matrix, α-cyano-4-hydroxycinnamic acid (CHCA; Merck, Germany), was then deposited on the slide using an iMLayer sublimation device (Shimadzu, Japan) for 10 minutes. It is worth mentioning that very recently, new techniques were introduced that enabled imaging in both modes with high sensitivity.^21^ After matrix application, MALDI IT-TOF MS/MS spot analysis was carried out in positive ionization mode using the iMScope Trio (Shimadzu, Japan) instrument with the following laser D2 settings: a laser diameter of 25 μm; pitch size of 22 × 20 μm; laser intensity of 46.8%; frequency of 20 Hz; 20 shots per pixel. The mass range was set to 150–380 Da, with a sample voltage of 3.5 kV and detector voltage of 1.9 kV. Conditions of the MS/MS analysis were as follows: the precursor ion was set to 337.10 ± 1.0 Da; the collision induced energy (CID) energy was set to 100%; the isolation time was set to 10 ms, while the rest of the parameters were set to their respective default values.

#### 2.3.2 MALDI IT-TOF MS^1^ imaging

MALDI IT-TOF MS analysis was conducted using the same MALDI IT-TOF MS device. CD19+ lymphocytes were localized on the sample using recorded coordinates obtained prior to matrix application. After taking a snapshot of the chosen cell using an integrated light microscope in the incident mode, the following analytical conditions were used in positive ionization mode. To capture a single cell, D1 settings were applied: 10 μm laser diameter; pitch size of 11 × 9 μm; 21.7% laser intensity, 20 Hz frequency, 20 shots per pixel and the mass range was set to 300–600 Da. Approximately 36 pixels were recorded for each CD19+ lymphocyte and its surroundings. A detailed description can be found in the work by Marković *et al.*^6^

#### 2.3.3 MALDI IT-TOF MS/MS imaging of CD19+ lymphocytes

Using the previously described experimental conditions, the intracellular content of the CD19+ lymphocytes, especially of those cell that did not release their contents during sublimation, was not desorbed completely. Due to the small amount of material desorbed from a single cell, the laser intensity was increased to 50%. To enhance sensitivity, the detector voltage was increased to 2.1 kV. Other settings used in the single-cell MS^1^ analysis were retained. In addition to the mass range and the precursor ion, all MS/MS conditions were set to the same values described in the previous section. To capture smaller fragments, the mass range was set to 100–380 Da, while the precursor ion was set to 337.11 ± 0.05 Da to avoid interference from endogenous compounds with similar *m*/*z* values.

#### 2.3.4 Analysis of the empirical data

All mass spectra and MS images were generated by ImageReveal software v.1.1. (Shimadzu, Japan). Statistical analysis of total-ion-current (TIC)-normalized spectra was analysed using the R statistical package v. 4.2.^22^ Statistical significance was set to 0.05.

### 2.4 *In silico* AIMD-based MS/MS spectra

To obtain the theoretical mass spectra of the observed molecules, each molecule was first protonated by screening the possible hydrogenation sites using the CREST V2.12 programme package.^23,24^ This conformational searching method alleviates the problems associated with traditional searching approaches, such as simulated annealing or brute force sampling methods, which are based on non-quantum mechanics.^25^ Although the simulation of the protonation process can involve complicated models, studies have shown that these difficulties arise when multiple protonation states are present.^26^ Screening was conducted with the extended tight-binding method GFN-xTB, which employs the density functional theory (DFT)-D4 model for the efficient computation of inter- and intramolecular dipole–dipole dispersion coefficients.^16^ Adducts of the selected molecules were relaxed at the DFT level with the ORCA V6.0.0 suite of programs.^27^ DFT calculations were conducted with the PBEh-3c correlation functional.^28^ While more accurate levels of theory exist, such as the coupled cluster extension to the Hartree–Fock method, this choice of the functional provided an optimal ratio of accuracy and simulation time requirements with respect to the available computational resources.^29^ The stability of the optimized structures was assessed by calculating the vibrational frequencies of the molecules and ensuring that no imaginary frequency modes existed. In the case of lysophosphatidic acid (8 : 0, 0 : 0) (LPA8) and 7,8-dihydropteroic acid (H2Pte), the same workflow was also applied using potassium and sodium, respectively, instead of hydrogen.

In the following step, CID spectra were calculated using the Born–Oppenheimer molecular dynamics (MD) as implemented in the QCxMS V5.2.1 programme package.^30,31^ The workflow consisted of: (i) equilibrating the protonated molecules using MD at 300 K to ensure canonical ensemble conditions (*NVT*), (ii) generating different starting geometries using the microcanonical ensemble (*NVE*), and (iii) simulating collision events using MD. Argon was used as the collision gas. Each starting geometry of the parent ion from the previous step corresponded to one run. In the calculations, 2000 runs were simulated for each case. MD simulations were performed with a time step of 0.5 fs. Throughout this study, the PlotMS utility^32^ was used to compute and visualize the resulting spectra.

## 3 Results and discussion

As a proof-of-concept for the metabolite annotation strategy, the problem from our previous study^6^ (presented in Fig. 1) was chosen: part of the single-cell MALDI IT-TOF MS^1^ mass spectra averaged over all analysed CD19+ lymphocytes from CLL patients and blood donor (BD) group is presented.

The metabolite database search using 337.11 Da yielded 5 different search hits (Table 1). Three of these hits were glutathione-related peptides, while the remaining 2 were unrelated compounds. Aside from these 5 hits, other hits corresponded to exogenous molecules that were disregarded. Furthermore, some database search hits were disregarded due to the formation of uncommon adducts or due to their biological role, which is not related to human lymphocytes.

According to the literature, AIMD-based fragmentation spectra have never been used for the annotation of *m*/*z* values from imaging experiments. This fact led us to question the suitability of AIMD-based fragmentation as a tool for interpreting MALDI IT-TOF MS/MS spectra.

**Fig. 2:**
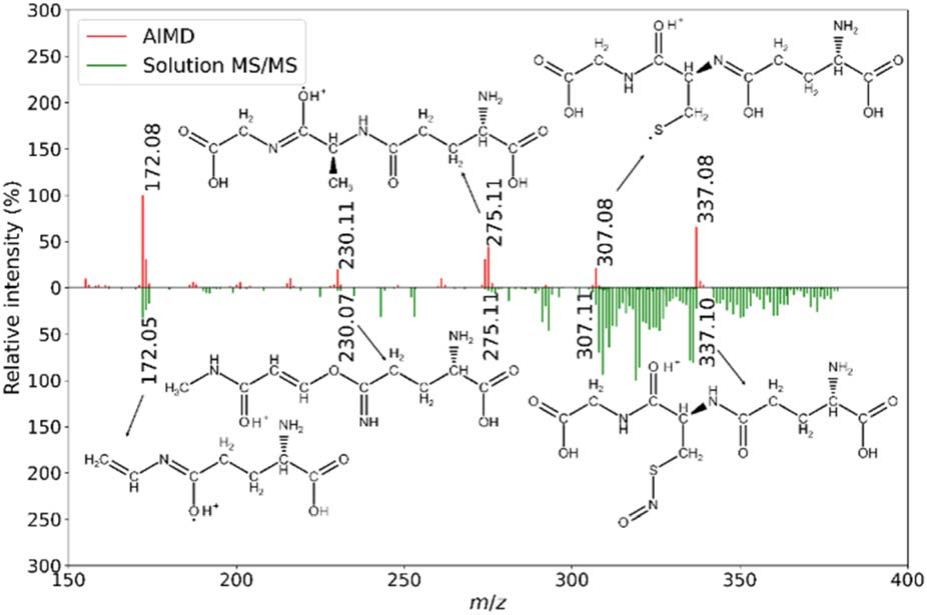
Comparison of the *in silico* AIMD-based MS/MS and empirical MS/MS spectra of GSNO. For each pair of matching lines, corresponding *m*/*z* values and 2D chemical structures are shown. Different tautomeric forms of the corresponding chemical structures are possible.

**Fig. 3:**
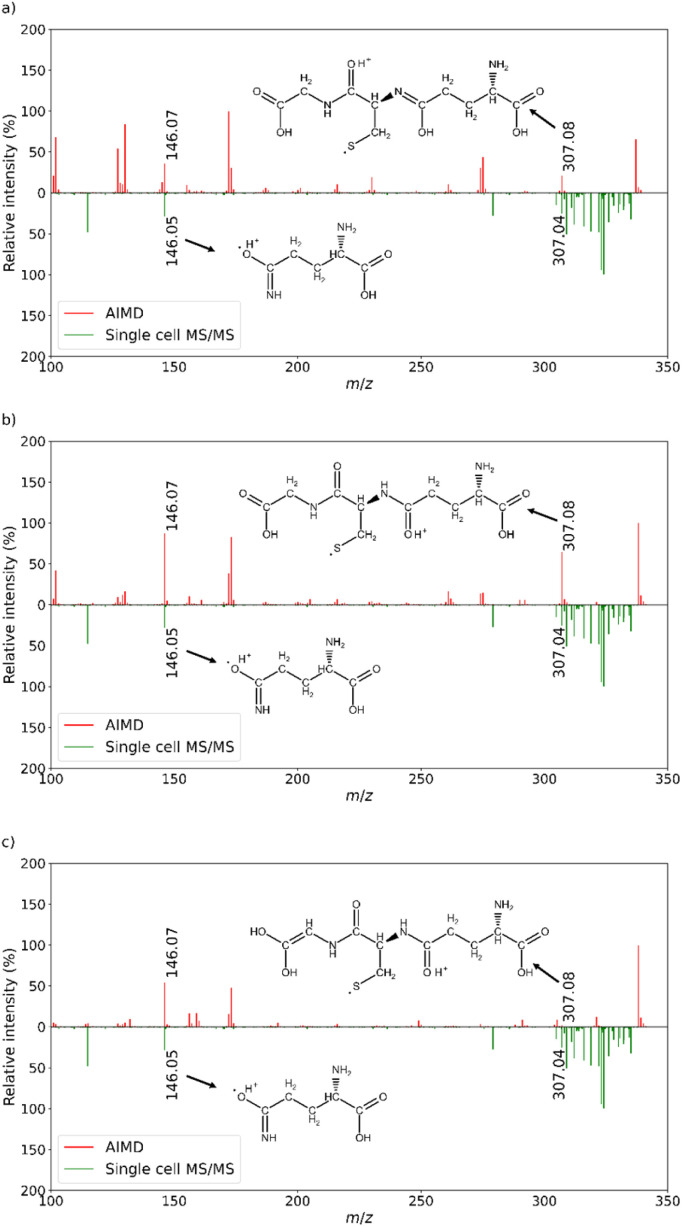
Comparison of the *in silico* AIMD-based MS/MS spectra of the tentatively annotated metabolites and empirical MS/MS spectrum of a selected lymphocyte. (a) GSNO; (b) GSHM; (c) γ-ECS. Protonated adducts were used to generate the AIMD-based fragmentation spectra. For each pair of matching lines, the corresponding *m*/*z* values and 2D chemical structures are shown. Different tautomeric forms of the corresponding chemical structures are possible. The *m*/*z* values the cluster in the 300–330 Da range also exist in the blank. All *m*/*z* signals associated with the blank experiment were excluded from the annotation process. (SI, Fig. S1).

Comparison of the empirical MS/MS spectra and *in silico* spectra of the GSNO solution (Fig. 2) provides evidence of the suitability, at least for the glutathione-related compounds: 5 fragments from the imaging and the theory matched to <0.05 Da. Furthermore, the theoretical and empirical relative intensities of these fragments were similar. These results paved the way for the MS/MS imaging experiments. The MS/MS imaging of the lymphocytes required minor adjustments of the instrumental settings used in MS^1^ imaging. The MS/MS spectra of a selected lymphocyte are given in Fig. 3.

The single-cell MALDI IT-TOF MS/MS analysis results were compared with those of the three glutathione-related tentatively annotated compounds: 2 fragments coming from GSNO, GSHM and γ-ECS were present in both the *in silico* and the empirical MS/MS spectrum (Fig. 3). The blank experiment conducted under the same conditions showed no significant signals at *m*/*z* values of the fragments (SI, Fig. S1). H2Pte and LPA8 produced no *m*/*z* values shared by the *in silico* and empirical MS/MS spectra (SI, Fig. S2–S5). The AIMD-based fragmentation of the sodium H2Pte adduct and potassium LPA8 adduct produced no significant amounts of fragments. Due to this finding, we conducted an AIMD calculation using protonated H2Pte and protonated LPA8. In all cases, no fragment matching was observed. It should be noted that, due to theoretical limitations, the *in silico* MS/MS spectra of GSHM and γ-ECS were also generated using their protonated forms, while other adduct types of the corresponding compounds were described in the original study (Table 1). In the case of GSHM and γ-ECS, this approximation produced a reasonably good match between the *in silico* and empirical observations.

When compared to the empirical spectrum of the GSNO solution, the single-cell MS/MS spectra showed some differences. The precursor ion and some fragment ions were not detected in the latter spectra, which might be attributed to the miniscule cellular content of GSNO, but it can also shift the annotation rationale towards other candidate compounds (Table 1). However, the 307.08 Da fragment existed in both the MS/MS spectra of the GSNO solution and single-cell MS/MS spectra. This fragment also existed in the AIMD-based fragmentation spectra derived from all the glutathione-related compounds (Table 1 and Fig. 3). This finding shows that even if the protonated adduct was used for the AIMD-based fragmentation, the calculated spectrum at least partially corresponded to the empirical MS/MS spectra produced for the other adduct type of the same molecules, namely γ-ECS and GSHM. It was also interesting to note that the AIMD calculations produced fragments with the same molecular formula but different connectivity. This finding suggests that an MS^3^ experiment using the fragment at 307.08 Da as a precursor may be useful for the differentiation of the given glutathione-related compounds. One more fragment at 146.07 Da, which according to theory is common to all glutathione-related compounds, was detected in the single-cell MS/MS experiment. This match makes classification of the 337.11 Da ion from MS/MS imaging to the glutathione-related compounds group more likely. Considering that the MS/MS spectrum of the blank sample showed no signals at 307.08 and 146.07 Da, the given classification is even more convincing (SI, Fig. S1).

Differentiation between the members of the class of glutathione-related compounds (GSNO, γ-ECS and GSHM) was not achieved by the qualitative fragmentation correspondence analysis of the lymphocytes. However, comparison of the fragment intensities obtained from the theoretical and empirical spectra may be useful, as suggested by comparing the results of the MS/MS spectra of the GSNO solution with the AIMD-based fragmentation spectra. According to the intensities in Fig. 3, the best correspondence between the *in silico* 307.08 Da and 146.07 Da fragment intensities and corresponding empirical intensities was obtained for GSNO. γ-ECS and GSHM show greater discrepancies for both fragments. Given that GSNO and GSHM belong to the same biochemical pathway, changes in their cellular contents should provide very similar biological information.^33^ However, distinction of GSNO and GSHM from the γ-ECS would be more interesting. In this regard, MS^3^ or ion-mobility experiments might have proved useful.

**Fig. 4:**
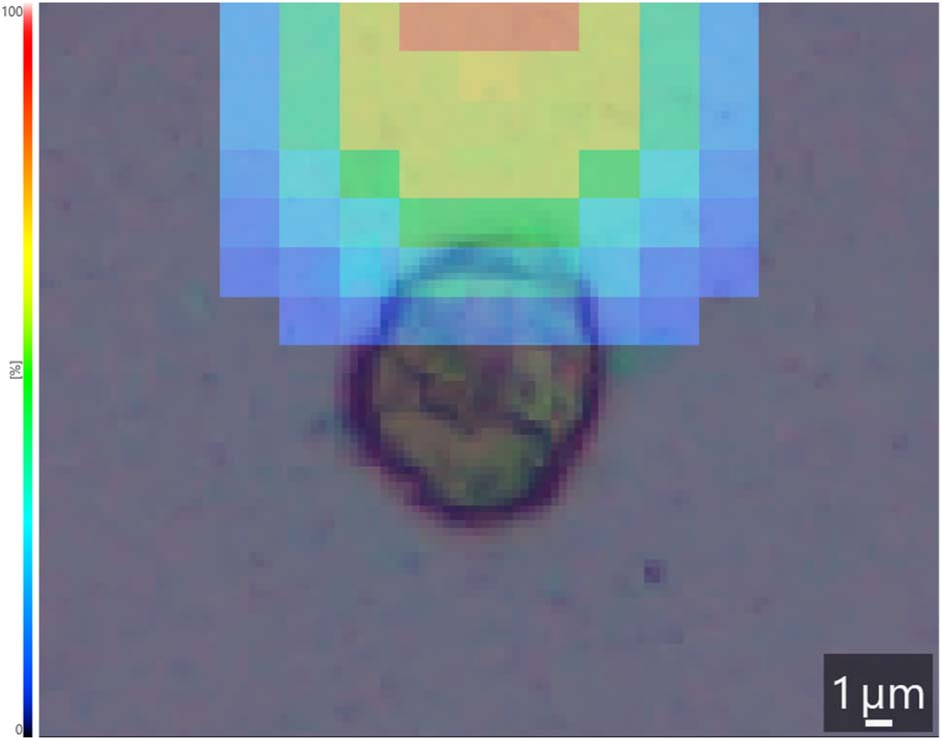
The lateral distribution of fragment ion (*m*/*z* 146.05 Da) intensities from the single-cell MS/MS spectrum shown in Fig. 3 overlaid on the optical-microscopy image of the respective non-stained lymphocyte. Relative fragment ion intensity is visualized by a colour scale on the left side of the image. No postanalytical image alignment was applied.

Adjustments of the instrumental variables required for the single-cell MS/MS experiments included an increased detector voltage and increased laser intensity. Under the given settings, clearly visible single-cell MS/MS images were produced for an isolated lymphocyte (Fig. 4).

The lateral distribution of the 146.05 Da fragment was used for generation of the MS image (Fig. 4). The image depicted at 1 μm resolution shows a shift of ∼3 μm between the lymphocyte and the recorded intensity distribution at *m*/*z* 146.05 Da. Besides the stronger fragment signals, the increased laser intensity enabled greater release of the intracellular contents, which might be useful even in the MS^1^ experiments. However, the increased laser intensity widened the laser spots and may have caused slight shifts in the MS/MS image (Fig. 4). To accommodate the wider laser pitch to the cellular diameter, the number of pixels per cell may be reduced, which is expected to improve the MSI and optical image alignment.

The focus of this study was to show that metabolite annotation using single-cell MALDI IT-TOF MS/MS imaging is possible. However, analytical and especially biological variation may significantly affect the conclusions. Fig. 1b shows the intensity distribution at 337.11 Da in the MS^1^ imaging of the CD19+ lymphocytes: the given image suggests great dispersion of the intensities, probably due to the variations in the metabolism of each analysed lymphocyte. This variation is reflected in the single-cell MS/MS experiments. During the development of the single-cell MS/MS imaging method, more than 20 CD19+ lymphocytes were analysed. Most of the single-cell MS/MS spectra exhibited a signal at 307.08 Da. However, the number of fragments present in the empirical spectra of the analysed lymphocytes that corresponded to fragments present in the *in silico* spectra varied from 0–3: for the MS/MS spectra comparison, a lymphocyte containing 2 fragments was used.

As concluding remarks, some limitations of this study should be pointed out. Firstly, only protonated adducts proved to be useful in the AIMD calculations. Therefore, modifications of the *in silico* approach are needed to account for the fact that other adduct types may arise from the MALDI source. In that regard, some experimental modifications may be useful. For example, the application of matrices that are more effective in terms of protonation would be helpful. Additionally, the relatively complex sample preparation involving a number of different chemicals may have caused chemical changes of the analytes and consequently led to the rise of chemically modified analytes that are very hard to annotate by MS/MS experiments. Therefore, further improvement of the sample preparation procedures is needed. The blank experiment (SI, Fig. S1) showed signals above 310 *m*/*z*. Since these *m*/*z* clusters even existed in the MS/MS of the GSNO solution, the most probable explanation would be that these clusters represent complex matrix adducts or instrumentation background. The ongoing research will address this issue. To validate the proposed method with a large sample of metabolites, more time-efficient, *in silico* simulations may be used. Methods developed around conceptual density functional theory (CDFT) based on nuclear reactivity descriptors have provided such possibilities.^34^ However, these methods still lack verification for more classes of compounds. Finally, the application of this approach for metabolite annotation should be evaluated on relatively large sets of patients and cells: such a study is underway.

## 4 Conclusions

The AIMD-based fragmentation spectra showed good correspondence with the empirical MS/MS spectra of the GSNO solution. This finding enabled the comparison of the *in silico* spectra and the single-cell MS/MS spectra of the CD19+ lymphocytes obtained from the CLL patient. MS/MS analysis of the CD19+ lymphocytes, using slightly adjusted instrumental conditions and 337.11 Da ion as a precursor, produced spectra useful for metabolite annotation. Based on the correspondence with the *in silico* fragmentation, the given *m*/*z* value was assigned to glutathione-related compounds, most likely GSNO. For exact annotation, MS^3^ or ion mobility experiments are needed. The lateral distribution of the selected fragment showed reasonable correspondence with the respective lymphocyte. Under the given instrumental conditions, the MS/MS imaging of different cell types in cell suspensions is expected to enable metabolite annotation by comparison with AIMD-based fragmentation.

## Author contributions

Mateo Topalović: investigation, data curation, formal analysis, visualization, writing – original draft; Ivana Marković: conceptualization, validation, writing – review and editing; Vlatka Periša: resources, writing – review and editing; Maja Lukić: investigation, writing – review and editing; Ema Pavičić: investigation, writing – review and editing; Iva Lukić: investigation, writing – review and editing; Stefan Mrđenović: resources, writing – review and editing; Igor Lukačević: formal analysis, investigation, supervision, funding acquisition; Željko Debeljak: conceptualization, methodology, writing – original draft, writing – review and editing, funding acquisition.

## Conflicts of interest

The authors declare that there are no conflicts to declare.

## Abbreviations

γ-ECS: γ-Glutamylcysteinylserine
AIMD: *Ab initio* molecular dynamics
CLL: Chronic lymphocytic leukemia
DFT: Density functional theory
GSHM: *S*-Hydroxymethylglutathione
GSNO: *S*-Nitrosoglutathione
H2Pte: 7,8-Dihydropteroic acid
IT: Ion trap
LC: Liquid chromatography
LPA8: Lysophosphatidic acid (8 : 0, 0 : 0)
MALDI: Matrix assisted laser desorption/ionization
MS: Mass spectrometry
MS/MS: Tandem mass spectrometry
MSI: Mass spectrometry imaging
TOF: Time-of-flight

## Supplementary Material

RA-015-D5RA05470B-s001

## Data Availability

The raw data used in the study will be freely available at https://repozitorij.kbco.hr/islandora/object/kbco%3Aclanci. Supplementary information is available. See DOI: https://doi.org/10.1039/d5ra05470b.
