# Supplementary material for: Single-cell metabolite annotation by tandem mass spectrometry imaging and ab initio molecular dynamics-based fragmentation
Source: RSC Adv. 2025 Sep 15;15(40):33515–21. doi: 10.1039/d5ra05470b (PMC12434585; doi:10.1039/d5ra05470b)
Supplement: RA-015-D5RA05470B-s001 [file RA-015-D5RA05470B-s001.pdf]

Supplementary Information (SI) for RSC Advances.
This journal is © The Royal Society of Chemistry 2025

Supplementary materials

for

Single-cell metabolite annotation by mass spectrometry imaging and *ab initio* molecular dynamics-based fragmentation

Authors:

Mateo Topalović, Ivana Marković, Vlatka Periša, Maja Lukić, Ema Pavičić, Iva Lukić, Stefan Mrđenović, Igor Lukačević, Željko Debeljak*

* Corresponding author

e-mail: zeljko.debeljak@gmail.com

Contents

1. Supplementary information 1 (SI1): The blank sample 3

2. Supplementary information 2 (SI2): 7,8-dihydropteroic acid 4

   2.1 Sodium adduct of 7,8-dihydropteroic acid 4

   2.2 Protonated 7,8-dihydropteroic acid 5

3. Supplementary information 3 (SI3): Lysophosphatidic acid (8:0, 0:0) 6

   3.1 Potassium adduct of Lysophosphatidic acid (8:0, 0:0) 6

   3.2 Protonated Lysophosphatidic acid (8:0, 0:0) 7

1. Supplementary information 1 (SI1): The blank sample

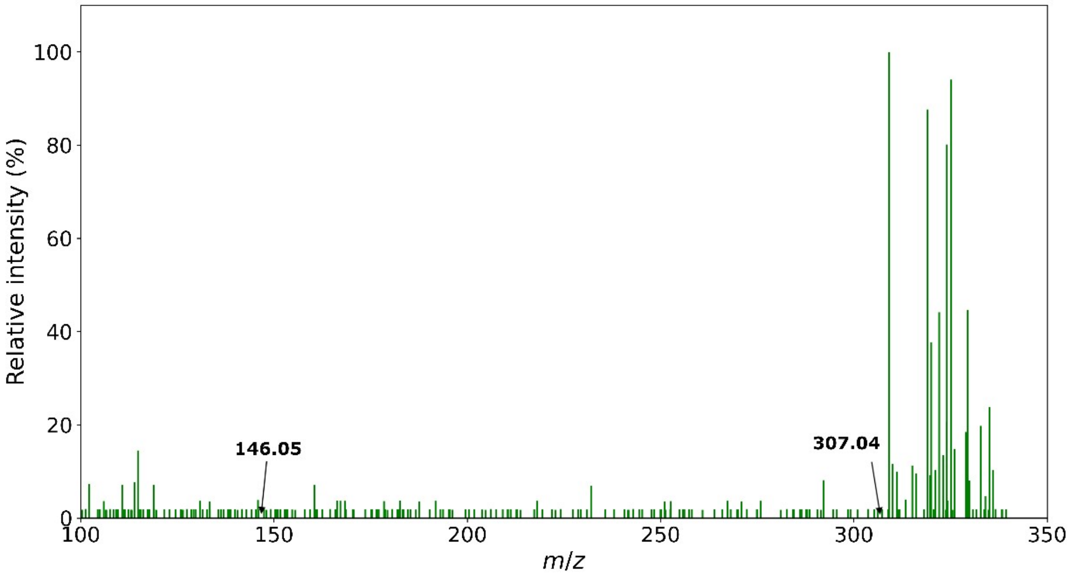

Figure S1. MS/MS spectrum of the blank sample. The experiment was conducted under the same conditions as the single cell experiment. MS/MS spectrum of the blank sample showed no signals at 146.05 and 307.04 m/z.

2. Supplementary information 2 (SI2): 7,8-dihydropteroic acid

2.1 Sodium adduct of 7,8-dihydropteroic acid

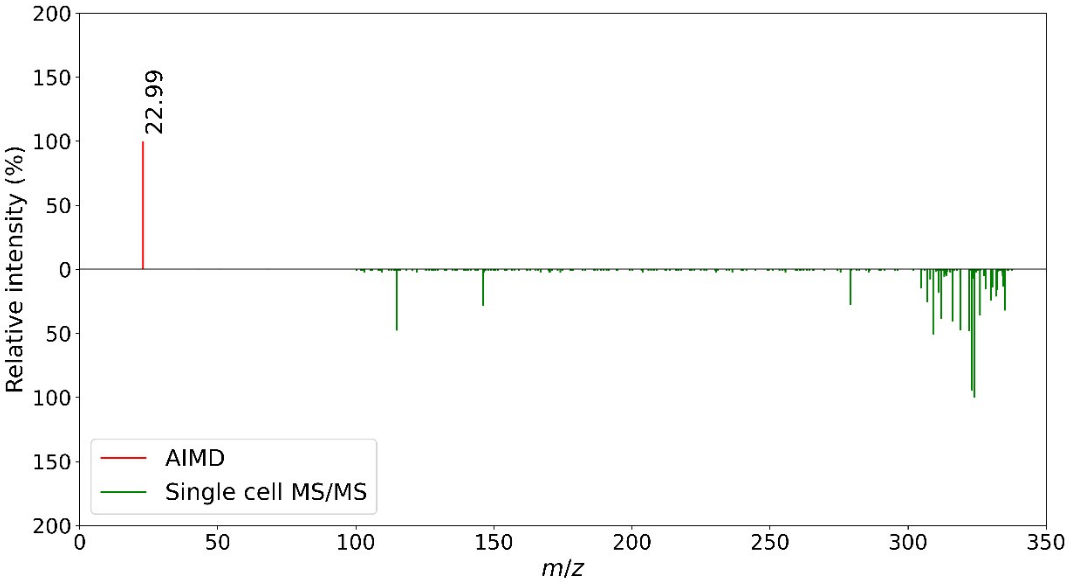

Figure S2. Comparison of the *in silico* AIMD-based MS/MS spectrum of 7,8-dihydropteroic acid sodium adduct and empirical single cell MS/MS spectrum.

2.2 Protonated 7,8-dihydropteroic acid

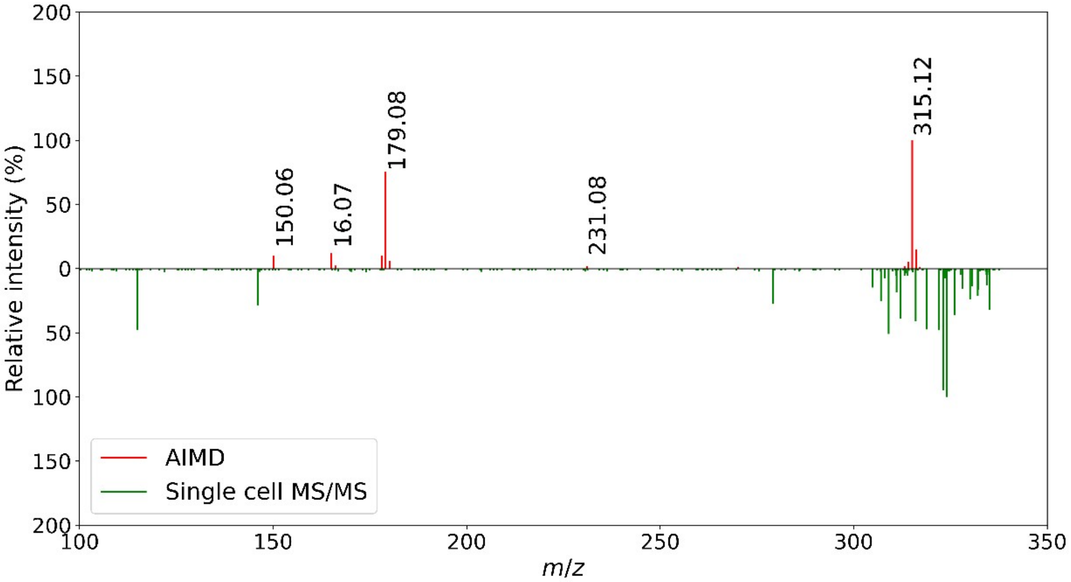

Figure S3. Comparison of the *in silico* AIMD-based MS/MS spectrum of protonated 7,8-dihydropteroic acid and empirical single cell MS/MS spectrum.

3. Supplementary information 3 (SI3): Lysophosphatidic acid (8:0, 0:0)

3.1 Potassium adduct of Lysophosphatidic acid (8:0, 0:0)

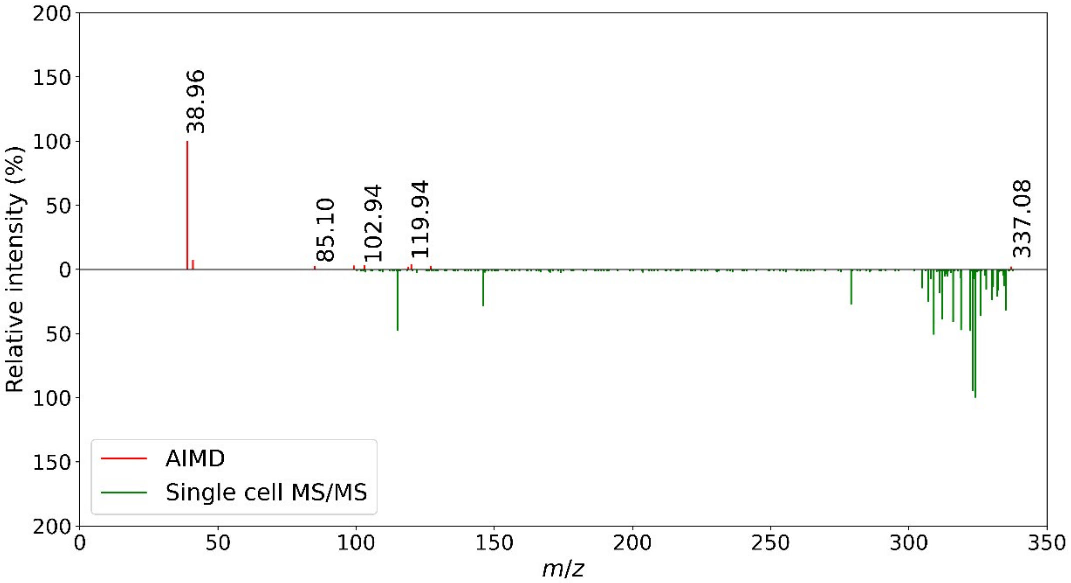

Figure S4. Comparison of the *in silico* AIMD-based MS/MS spectrum of Lysophosphatidic acid (8:0, 0:0) potassium adduct and empirical single cell MS/MS spectrum.

3.2 Protonated Lysophosphatidic acid (8:0, 0:0)

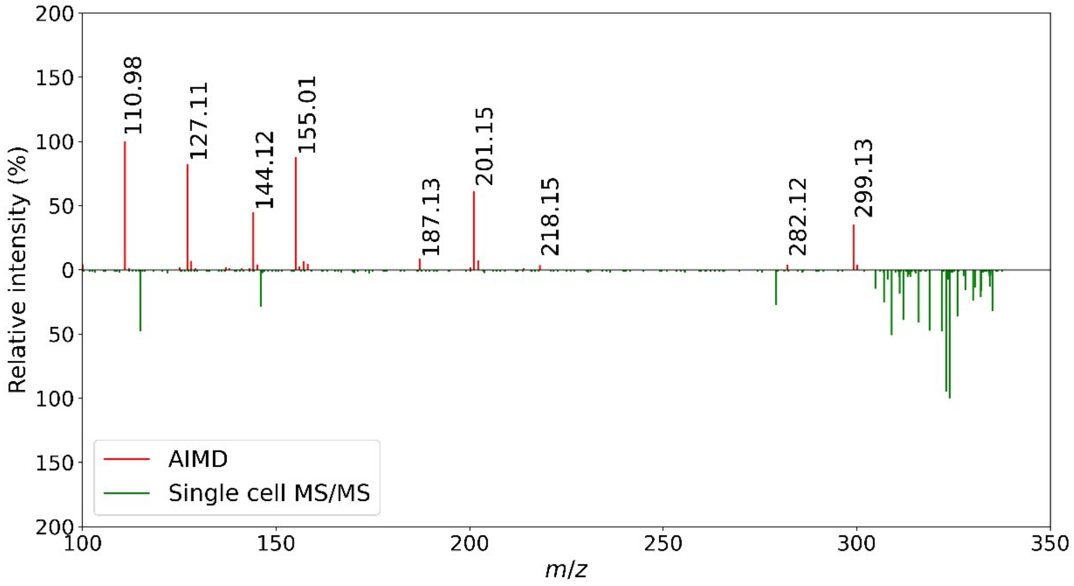

Figure S5. Comparison of the *in silico* AIMD-based MS/MS spectrum of protonated Lysophosphatidic acid (8:0, 0:0) and empirical single cell MS/MS spectrum.
